# Addressing Anxiety and Stress for Healthier Eating in Teens (ASSET): A Pilot Randomized Controlled Trial Protocol for Reducing Anxiety, Disinhibited Eating, Excess Weight Gain, and Cardiometabolic Risk in Adolescent Girls

**DOI:** 10.3390/nu14204246

**Published:** 2022-10-12

**Authors:** Hannah E. Repke, Lauren D. Gulley, Alexander J. Rice, Julia H. Gallagher-Teske, Bethelhem Markos, Natalia Sanchez, Madison Bristol, Hannah Haynes, Jason M. Lavender, Mary K. Higgins Neyland, Lisa M. Shank, Jill E. Emerick, Ana M. Gutierrez-Colina, Thomas Arnold, Victoria Thomas, Mark C. Haigney, Lauren B. Shomaker, Marian Tanofsky-Kraff

**Affiliations:** 1 Military Cardiovascular Outcomes Research (MiCOR) Program, Department of Medicine, Uniformed Services University, Bethesda, MD 20814, USA; 2 The Metis Foundation, San Antonio, TX 78216, USA; 3 Department of Human Development and Family Studies, Colorado State University, Fort Collins, CO 80523, USA; 4 Department of Pediatrics, Section of Endocrinology, University of Colorado Anschutz and Children’s Hospital Colorado, Aurora, CO 80045, USA; 5 Colorado School of Public Health, Aurora, CO 80045, USA; 6 Department of Medical and Clinical Psychology, Uniformed Services University, Bethesda, MD 20814, USA; 7 Department of Pediatrics, Uniformed Services University, Bethesda, MD 20814, USA

**Keywords:** anxiety, interpersonal psychotherapy, cognitive behavioral therapy, disinhibited eating, excess weight gain prevention, cardiometabolic risk

## Abstract

(1) Background: Standard-of-care lifestyle interventions show insufficient effectiveness for the prevention and treatment of excess weight and its associated cardiometabolic health concerns in adolescents, necessitating more targeted preventative approaches. Anxiety symptoms are common among adolescents, especially girls at risk for excess weight gain, and have been implicated in the onset and maintenance of disinhibited eating. Thus, decreasing elevated anxiety in this subset of adolescent girls may offer a targeted approach to mitigating disinhibited eating and excess weight gain to prevent future cardiometabolic health problems. (2) Methods: The current paper describes the protocol for a multisite pilot and feasibility randomized controlled trial of group cognitive behavioral therapy (CBT) and group interpersonal psychotherapy (IPT) in *N* = 40 adolescent girls (age 12–17 years) with elevated anxiety symptoms and body mass index (BMI; kg/m^2^) ≥ 75th percentile for age/sex. (3) Results: Primary outcomes are multisite feasibility of recruitment, protocol procedures, and data collection, intervention fidelity, retention at follow-ups, and acceptability of interventions and study participation. (4) Conclusions: Findings will inform the protocol for a future fully-powered multisite randomized controlled trial to compare CBT and IPT efficacy for reducing excess weight gain and preventing adverse cardiometabolic trajectories, as well as to evaluate theoretically-informed treatment moderators and mediators.

## 1. Introduction

The prevalence of overweight (body mass index (BMI) kg/m^2^ ≥ 85th percentile) and obesity (BMI ≥ 95th percentile) [1] in adolescents represents a major public health concern [2], as approximately 35% of adolescents in the United States have overweight or obesity. Excess weight in adolescence tends to persist into adulthood [3,4,5,6] and increases risk for immediate and long-term cardiometabolic health concerns [7], including hypertension, type 2 diabetes, and cardiovascular disease [8,9,10,11,12,13].

Adolescence is a dynamic stage of life characterized by interacting changes in psychosocial and physical health domains [14]. As such, adolescence is a critical period of overall development and may represent a particularly impactful window for the prevention of excess weight gain and its sequelae [14]. Standard lifestyle interventions that focus primarily on promoting healthier eating and increasing physical activity for the prevention or treatment of excess weight have shown insufficient efficacy, particularly in adolescents [15,16,17]. Instead, interventions targeting specific vulnerabilities underlying health-related behaviors during adolescence that affect weight and cardiometabolic health trajectories may offer a more promising approach [15,18]. Disinhibited eating and anxiety, two related constructs discussed in the ensuing sections, may serve as promising intervention targets in the prevention of excess weight gain in adolescent girls.

### 1.1. Disinhibited Eating

Disinhibited eating refers to a range of behaviors characterized by a lack of restraint over what or how much is consumed [19]. Disinhibited eating increases in prevalence during the adolescent years, and thus is a health-related behavior of particular pertinence to weight and cardiometabolic health in adolescence [19]. Disinhibited eating may manifest in a number of ways, but one of the most prevalent forms among adolescent girls with excess weight is loss of control (LOC) eating [20,21], or the subjective experience of being unable to stop or control how much one is eating, regardless of the amount of food consumed [22]. LOC eating overlaps with other types of disinhibited eating, including emotional eating (i.e., eating in response to certain emotional experiences) and eating in the absence of hunger (i.e., eating in response to cues other than physiological hunger, such as boredom) [19,23].

A propensity for disinhibited eating, including LOC eating, is associated cross-sectionally with excess weight and adiposity among children and adolescents [24]. In prospective studies, youth reporting disinhibited eating have been found to be at heightened risk for excess weight gain, adiposity gain, and development of metabolic syndrome [25,26,27,28]. Disinhibited eating among children and adolescents has also been related to cardiometabolic health concerns, including higher systolic blood pressure and more elevated low-density lipoprotein (LDL) cholesterol [29], insulin resistance [24], elevated heart rate and decreased heart-rate variability [30], and greater inflammation [31]. Together, these outcomes may place adolescents with a propensity for disinhibited eating at heightened risk for developing preventable chronic diseases, including hypertension, type 2 diabetes, and cardiovascular disease [9,10,11,12].

### 1.2. Anxiety Symptoms and Disinhibited Eating

Although multiple factors are believed to contribute to the development and persistence of disinhibited eating, e.g., [19,32], the role of negative affective experiences in particular has been highlighted e.g., affect theory [33]; this role has been supported in studies of adolescents, including those with excess weight [34]. Anxiety appears to be a particularly salient form of negative affect with regard to the onset and maintenance of disinhibited eating episodes in adolescents e.g., [24]. For example, in an investigation of adolescent girls’ pre-prandial negative affective states during a laboratory test meal designed to simulate a disinhibited eating episode, anxiety was associated with higher food intake, but no significant associations were found for depression, fatigue, confusion, or anger and food intake [35].

Elevated anxiety symptoms are common in adolescents [36], especially among those with excess weight [37]. Meta-analytic data show a positive association between anxiety symptoms and BMI in youth, and this link may be stronger among females compared to males [38]. Anxiety symptoms have also been related to greater adiposity in adolescent girls [39]. It is possible that symptoms of anxiety promote excess weight and adiposity gain through a variety of stress-related behavioral and physiological mechanisms, with disinhibited eating being one strong candidate as an explanatory behavior [40]. In a cross-sectional study of children and adolescents, anxiety symptoms were found to be indirectly associated with both BMI and adiposity via the number of recent LOC episodes [41]. Furthermore, among adolescent girls, physiological indicators of stress, including increased heart rate and decreased heart rate variability [42], have been shown to predict the real-time experience of disinhibited eating episodes [30]. While anxiety also impacts boys, given the robust literature supporting links between anxiety, adiposity, and cardiometabolic functioning in girls, an initial pilot study among females only is warranted.

Importantly, anxiety symptoms are also associated with poorer metabolic health across the lifespan, even when accounting for BMI [43]. Disinhibited eating may be one explanatory factor in the associations of anxiety symptoms with excess weight and cardiometabolic risk indicators among adolescents. For example, among adolescent girls with high anxiety symptoms, a positive association between anxiety symptoms and insulin resistance was found only among those with LOC eating [24].

### 1.3. Targeting Anxiety to Reduce Disinhibited Eating/Excess Weight Gain

Given the potential role of anxiety in promoting disinhibited eating and its downstream negative effects on excess weight and cardiometabolic health, reducing anxiety in adolescents at risk for excess weight gain may represent a targeted approach to mitigating disinhibited eating and preventing excess weight gain and worsening of cardiometabolic health. Average effect sizes for the treatment of anxiety in adolescents (Cohen’s *d* = 0.39–0.76) are generally higher than those for treatment of obesity in adolescents (Cohen’s *d* = 0.13) [44,45]. Individual and group-based delivery modes appear to be equally effective for anxiety in adolescents [46]. However, group-based formats offer the advantages of built-in exposure to social interactions with peers, which itself may provide a therapeutic mechanism for decreasing anxiety [47], and group delivery is typically more cost-effective than individual approaches.

Importantly, there has been less research on group delivery via telehealth, a format that has become much more common in practice since the onset of the COVID-19 pandemic [48,49]. Telehealth group delivery holds the prospect of increasing access for more adolescents, which is particularly important at a time when mental health concerns, including anxiety, are at an all-time high [50], paralleling spikes in excess weight gain [51] and adolescent-onset of type 2 diabetes [52].

Two group therapies, cognitive behavioral therapy (CBT) and interpersonal psychotherapy (IPT), may be suitable for reducing anxiety and disinhibited eating in adolescents at risk for excess weight gain. Both CBT and IPT have been shown to effectively reduce binge-eating episodes in adults [53,54,55,56,57,58]. In adolescents, CBT is a standard-of-care approach for the prevention and the treatment of anxiety [59]. Conversely, adolescents with elevated anxiety appear to be highly responsive to decreasing depressive symptoms in response to IPT [60,61] and to gaining less excess weight and adiposity over time following IPT [56]. In a randomized controlled trial comparing group IPT to a didactic health education control group in adolescent girls at risk for excess weight gain and LOC eating, few differences were found in BMI and adiposity trajectories at 1-year follow-up [62]. However, three years later, girls with higher baseline anxiety symptoms who had been randomized to IPT, versus health education, experienced the greatest reductions in BMI [56]. Moreover, girls with higher baseline anxiety symptoms in IPT did not experience adiposity gain, compared to significant adiposity gain in health education [56]. Furthermore, post-intervention remission from disinhibited eating was associated with girls’ improvements in cardiometabolic markers 6 months later [63], supporting the notion of targeting disinhibited eating for improving cardiometabolic health.

#### 1.3.1. Cognitive Behavioral Therapy (CBT)

CBT is based on cognitive behavioral theory, which posits that an individual’s anxiety derives from an interaction of negative thoughts and behavioral avoidance [64]. Maladaptive responses to anxiety, such as disinhibited eating, are conceptualized as being learned, and thus can be unlearned and replaced with healthier and more adaptive coping mechanisms [65]. As such, CBT targets both the negative thought and the behavioral avoidance patterns that reinforce and maintain anxiety symptoms. Cognitive strategies focus on identifying and restructuring negative thoughts and attitudes. Behavioral strategies include gradual exposure to anxiety-provoking stimuli and positive self-guided behavioral reinforcement.

#### 1.3.2. Interpersonal Psychotherapy (IPT)

IPT is grounded in interpersonal theory, which posits that a person’s affect is connected to their social relationships [66,67]. IPT therefore addresses the social problems that precipitate, maintain, and exacerbate negative affective experiences, including anxiety. Among adolescents, disinhibited eating, anxiety symptoms, and excess weight have all been associated with more difficulties in social functioning [68,69,70]. Thus, it is not surprising that the data support interventions that target and improve social functioning as a means of preventing excess weight gain and cardiometabolic risk [71]. To address disinhibited eating in adolescents, IPT focuses on managing conflict and increasing social support in order to reduce negative affect, and therefore decrease the consequential disinhibited eating, excess weight gain, and cardiometabolic concerns [72].

### 1.4. Study Objectives

**Figure 1:**
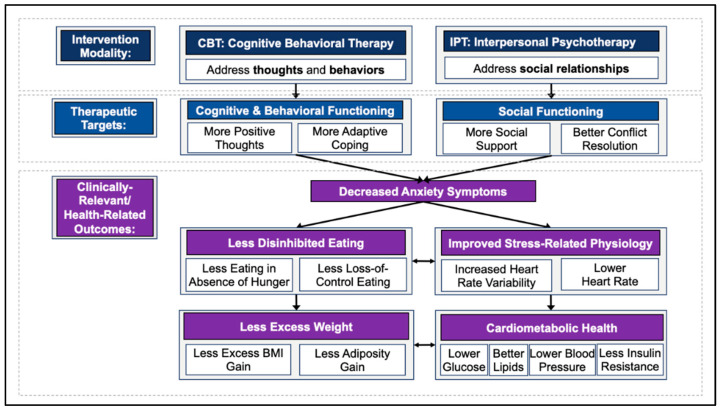
Theoretical model guiding the intervention targets and chosen relevant outcomes.

CBT and IPT both show promise in potentially impacting weight-related health trajectories and cardiometabolic outcomes among girls with elevated anxiety symptoms who are at risk for excess weight gain [56]. Our guiding conceptual model (Figure 1) is that CBT and IPT will both reduce anxiety symptoms, consequently decreasing disinhibited eating and improving stress-related physiology, which, in turn, will lead to prevention of excess weight gain and worsening cardiometabolic health over time. However, the therapeutic target for reducing anxiety is anticipated to differ in CBT (cognitive and behavioral functioning) versus IPT (social functioning). There is no study, as of yet, comparing CBT and IPT in ameliorating weight and cardiometabolic health trajectories among youth with elevated anxiety symptoms and at risk for excess weight gain.

The current protocol is a multisite pilot and feasibility randomized controlled trial of CBT and IPT in adolescent girls with elevated anxiety symptoms who are at risk for excess weight gain (Clinical Trials Identifier: NCT05038033). Multisite studies are ideal for maximizing external validity, timely recruitment of a targeted sample, and capacity for long-term follow-up [73]. Thus, to provide the best preliminary data to inform a future adequately powered multisite efficacy study, the primary aims of this multisite pilot study are to test feasibility of recruitment, feasibility of the study protocol and data collection, intervention fidelity, retention to follow-up assessments, and the acceptability of interventions and study participation. The secondary aim is to summarize changes in therapeutic targets (e.g., cognitive, behavioral, and social functioning), anxiety and depression symptoms, BMI and adiposity, and cardiometabolic health indicators, such as glucose levels, cholesterol, blood pressure, and insulin resistance.

## 2. Materials and Methods

Enrollment for this multisite pilot and feasibility randomized controlled trial was carried out at two sites: (1) the Uniformed Services University (USU) in Bethesda, Maryland and (2) Colorado State University (CSU) in Fort Collins, Colorado. Participants were 12- to 17-year-old adolescent girls with elevated anxiety symptoms as indicated by a total score of 32 or higher on the State-Trait Anxiety Inventory for Children (STAI-C) [74] and with a BMI ≥ 75th percentile for age/sex. Exclusion criteria were any major medical conditions including pregnancy or breastfeeding, regular medication use likely to impact mood or weight, current involvement in psychotherapy, and a psychiatric disorder or symptoms that, in the opinion of the investigators, would impede competence, compliance, or otherwise hinder completion of the study.

Recruitment methods included sending letters and flyers to parents of 12- to 17-year-old girls who resided near the respective sites; mailings to local physician offices that saw adolescent patients; advertising on school and parent email listservs, in local newspapers, and on social media (e.g., Facebook); and reaching out to families from previous studies who had expressed an interest in being contacted for future research. After accounting for attrition due to ineligibility, lack of interest, limited availability, and other circumstances, the target sample size was a total of *N* = 40 enrollment, with *n* = 20 per site. This sample size was in line with recommendations for pilot studies in which the primary effect size in a future efficacy trial is estimated to be small-to-moderate [75].

### 2.1. Clinically Relevant and Health-Related Measures

#### 2.1.1. Weight and Body Composition

Height was measured in triplicate with a stadiometer (Charder Electronic COLTD; Model HM 200P) and averaged. Fasting weight was measured on a calibrated digital scale connected to a Bod Pod (COSMED, Rome, IT). Height and weight measurements were used to calculate BMI indices [76]. A Bod Pod measures body composition using air displacement plethysmography [77]. Participants completed the Bod Pod measurement in a fasted state, wearing minimal clothing (spandex shorts or swimsuit) and a swim cap. The Bod Pod measured the participants’ body mass, body volume, and thoracic lung volume to calculate their body density and body fat percentage using the Siri [78] equation. A trained research staff member also measured the participants’ waist circumference with a tape measure (Gulick II) in triplicate and obtained an average for an accurate measurement.

#### 2.1.2. Cardiometabolic Health

Participants underwent phlebotomy in a fasted state. Two 5 mL EDTA tubes were collected (total 5.5 mL) to measure fasting glucose, insulin, glycated hemoglobin (A1c), LDL and HDL cholesterol, and other blood lipids. Fasting glucose was recorded as an average of two glucose measurements (Nova Biomedical, Waltham, MA, USA;; StatStrip Xpress2 Glucose Meter) taken immediately after the blood draw. The remaining blood was then processed and stored, and later analyzed in a Quest laboratory to measure the remaining blood values (e.g., insulin, A1c, cholesterol, and blood lipids). Fasting blood glucose and insulin were used to calculate insulin sensitivity and resistance using the quantitative insulin sensitivity check index (QUICKI) [79] and the homeostasis model assessment of insulin resistance (HOMA-IR) [80]. Resting blood pressure was also measured in triplicate with an automatic blood pressure monitor (LifeSource, Mississauga, Ont, CA; Model UA-789AC), and then averaged. The participants also wore a continuous glucose monitor (CGM; Abbott Laboratories Chicago, IL, USA; FreeStyle Libre Pro,) on the back of their upper arm for 7 days. The CGM was placed by a trained staff member and measured ambulatory glycemic patterns. Using this data, average daily glucose, peak glucose, standard deviation, and mean amplitude of glycemic excursions (all increases/decreases >1 SD of all values) were calculated to assess glucose homeostasis and glycemic variability, a marker and risk factor for type 2 diabetes [81].

#### 2.1.3. Stress-Related Physiology

The Carnation Ambulatory Monitor (CAM; Bardy Diagnostics, Seattle, WA, USA) was applied to the participant’s chest by a trained staff member and remained there for 7 days. The CAM captured electrocardiogram (ECG) signal and was used to measure heart rate and heart rate variability. The CAM data were also used to calculate the minimum, maximum, and mean heart rate, in addition to the PR interval (time between atrial activation to ventricular activation), QRS complex (duration of ventricular depolarization), QT interval (time between ventricular depolarization and repolarization), and QTc interval (QT interval corrected for heart rate). Standard deviation of normal beats and QTc interval variability, an indicator of stress and known risk factor for cardiovascular disease, were calculated to assess cardiac functioning and health.

#### 2.1.4. Daily Physical Activity

Participants wore the Movement and Activity Monitoring Device (activPAL; PAL Technologies Ltd. Glasgow, SCT, UK) on their upper thigh for 7 consecutive days. The activPAL uses an accelerometer to measure the participant’s position (lying, sitting, standing) and activity (active or sedentary) in 20-s increments. It then records the number of steps, stepping rate (i.e., cadence), and time in upright versus sedentary positions at each interval and as a daily and weekly average. The proprietary activPAL program used the raw data to calculate an activity summary score based on the amount of time active or sedentary and the intensity of activity.

#### 2.1.5. Disinhibited Eating

Several measures were used to assess various forms of disinhibited eating. The Eating Disorder Examination-Overeating Section (EDE-O) [82] is a semi-structured interview that identifies the frequency of disinhibited eating, specifically overeating and LOC eating, and has shown excellent interrater reliability with adolescents [83]. The Eating Disorder Assessment for DSM-5 (EDA-5) [84] is a computerized structured interview that assess the DSM-5 criteria for feeding and eating disorders (e.g., anorexia nervosa, bulimia nervosa, binge-eating disorder, and pica). The Emotional Eating Scale-Children (EES-C) [23] is a 25-item self-report measure used to assess the urge to cope with negative affect by eating. It generates three subscales: Anger/Anxiety/Frustration, Depression, and Unsettled, as well as a total score. The EES-C has demonstrated good internal consistency, discriminant validity [23], and construct validity [85]. The Eating in the Absence of Hunger Questionnaire for Children-Parent Report External Subscale (EAH-C-PR) is designed to measure parents’ perception of their child’s eating past satiation in response to external cues (e.g., how often does your child keep eating because others are still eating?) and negative affect (e.g., how often does your child keep eating because your child is feeling sad or depressed, nervous, angry, etc.?). The EAH-C-PR has demonstrated good construct validity in relation to food intake during an eating in the absence of hunger test-meal paradigm among adolescents [86].

#### 2.1.6. Anxiety and Depression Symptoms

The Social Phobia and Anxiety Inventory for Children (SPAI-C) is a 26-item self-report measure used to assess the somatic, cognitive, and behavioral aspects of social anxiety, with a higher total score indicating more anxiety symptoms. The SPAI-C has been validated in children aged 8–17 years old [87] and demonstrates good internal consistency, test-retest reliability, and discriminant and convergent validity [87,88]. The Center for Epidemiologic Studies-Depression Scale (CES-D) [89] is a 20-item self-report measure, with a higher total score indicating more depression symptoms. The CES-D has been shown to be reliable and valid in samples of adolescents, and confirmatory factor analysis suggests that the standard scoring of the CES-D with a total score is justified in adolescents e.g., [90].

#### 2.1.7. Ecological Momentary Assessments

Participants completed ecological momentary assessments for 7 consecutive days via the LifeData system (LifeData, LLC, Marion, IN, USA) and the associated mobile app available on iOS and Android platforms. Participants were able use their own smartphone or were given an iPod to complete the surveys. On weekdays, participants received three semi-random signals after standard school hours, and on weekends they received five semi-random signals during typical waking hours. Participants were prompted to answer questions about their current and recent (i.e., since their last report) negative affect (i.e., anxiety and depression symptoms), stress, disinhibited eating, avoidance of feared stimuli, negative cognitions, and perceived social threat. An additional signal was delivered at the end of the day that included questions about events and behaviors that could have influenced data collected from the monitoring devices (i.e., CAM, CGM, and activPAL).

### 2.2. Therapeutic Targets

#### 2.2.1. Cognitive and Behavioral Functioning

The Children’s Automatic Thoughts Scale—Negative/Positive (CATS-N/P) is a 50-item scale used to assess positive and negative thoughts in children and adolescents regarding internalizing and externalizing problems [91]. It includes four subscales regarding physical threat, social threat, failure, hostility, and positive thoughts. This measure has demonstrated good internal reliability and convergent validity in children and adolescents [91]. The Children’s Coping Strategies Checklist-Revised (CCSC-R1) [92] is a 54-item self-report measure with 13 subscales used to describe children’s coping efforts. The measure is reliable and valid in children and adolescents [93]. Finally, the Anxiety Control Questionnaire for Children (ACQ-C) [94] is a 30-item questionnaire used to measure children’s level of perceived control in anxiety related events. The measure has two subscales, one that measures perceived control over external threats and one that assesses anxiety-related internal bodily reactions. Adequate internal consistency has been supported and retest reliability is acceptable with children and adolescents [95].

#### 2.2.2. Social Functioning

The Social Adjustment Scale-Self Report (SAS) [96] is a widely used instrument assessing change in social functioning in three domains: school, friends, and family. The measure has demonstrated high internal consistency and test-retest stability [97] and is sensitive to change in social adjustment [98]. Additionally, the Network of Relationships Inventory-Short Form (NRI-Short Form) [99] assesses the extent to which adolescents’ dyadic relationships with parents, siblings, friends, romantic partners, and teachers are characterized by support or negative interactions. The NRI-Short Form is a 13-item measure used to measure relationships characteristics. The scale showed to have sufficient variability and the internal consistencies of the scales and factors were all adequate among children and adolescents [99]. Lastly, the Conflict Behavior Questionnaire (CBQ-20) [100,101] is used to measure a child’s conflict behavior separately with mother and father. The short form includes 20-items from the long form of the Conflict Behavior Questionnaire (CBQ-44), and is highly correlated with the CBQ-44, as well as being reliable and valid in children and adolescents [100].

#### 2.2.3. Affect and Emotion Regulation

The Positive and Negative Affect Schedule-Child Form (PANAS-C) [102] is a 30-item questionnaire used to measure the frequency of positive affect (e.g., interested, happy, excited,) and negative affect (e.g., sad, scared, mad). Adequate internal consistency has been supported for both the positive and negative affect scales [103]. The Difficulties in Emotion Regulation Scale-Short Form (DERS-SF) [104] is an 18-item measure of various dimensions of emotional dysregulation. The measure provides a total score and six subscale scores: non-acceptance of emotional responses; difficulty engaging in goal-directed behavior when distressed; impulse control difficulties when distressed; lack of emotional awareness; limited access to emotion regulation strategies; and lack of emotional clarity. The DERS-SF is highly correlated with the original 36-item DERS [105] and demonstrates good reliability and validity in adolescents [104].

#### 2.2.4. Self-Efficacy

The Self-Efficacy Questionnaire for Children (SEQ-C) [106] is a 24-item scale that measures adolescents’ beliefs about their competencies in three domains of self-efficacy: social (perceived capability for peer relationships and assertiveness), emotional (capability of coping with negative feelings), and academic (perceived capability to manage learning and academic expectations. Acceptable internal consistency has been found among children and adolescents [106]. SEQ-C subscales were used to evaluate both cognitive-behavioral functioning and social functioning therapeutic targets.

### 2.3. Procedures and Participant Timeline

#### 2.3.1. Pre-Screening Assessment

A trained research staff member pre-screened the adolescents and their parent/guardian who were interested in participating in the study over the phone to estimate their eligibility. The prescreening assessment occurred over the phone to maximize the likelihood that adolescents who came to the in-person screening and baseline at the research facility would be eligible to participate. The pre-screening included assessment of sex, age, height, and weight to estimate BMI, medication status, and general health for eligibility. Additionally, the adolescents completed surveys via a secure electronic data-capture system (e.g., Research Electronic Data Capture (REDCap)) to assess the inclusion criterion of elevated anxiety symptoms (i.e., total score ≥ 32 on the STAI-C) [74]. Adolescents who were interested and appeared to be eligible were scheduled for an in-person screening and baseline data collection visit.

Prior to any data collection, the study team member completed the consent and assent process electronically over a secure video conference platform, where the interested adolescents and their parent/guardian provided electronic assent and consent, respectively, using REDCap. Adolescents and their parents were informed of the possible risks and inconveniences of the study, as well as their right to withdraw at any time without penalty. Families were asked to bring a signed copy of the consent form to their first visit or, if they preferred to complete the consent/assent process in-person, they could do so before beginning the first visit at the research facility.

#### 2.3.2. Screening/Baseline Assessment

For a complete and detailed participant timeline, including the timing of all assessments, see Table 1. The screening visit involved an approximately 4-h morning appointment following an overnight fast to collect fasting weight and blood. The participant’s height and weight were measured and used to calculate BMI and determine inclusion on the basis of BMI ≥75th percentile. The participant’s parent/guardian completed a brief psychiatric and medical health history form about the participant and their family with a trained staff member to ensure the adolescent’s good general health and to re-check medication status.

A member of the research team administered the interviews to participants, with questionnaires administered via REDCap. These measures assess disinhibited eating, internalizing symptoms, cognitive and behavioral functioning, social functioning, affect, and emotional regulation. Parents/guardians also completed questionnaires via REDCap that assessed their child’s demographic information, social functioning, and eating patterns.

#### 2.3.3. Baseline Real-Time, Real-World Data Collection

At the end of the screening appointment, a trained staff member applied three monitoring devices that assessed daily glucose, heart rate, and physical activity/sedentary time. They were asked to wear the devices for 7 consecutive days. During the same 7-day period, participants were instructed to complete ecological momentary assessments on their mobile electronic device.

#### 2.3.4. The 12-Week Group Telehealth Intervention

Individuals who were determined to be eligible upon completion of the screening/baseline assessment were randomly assigned to CBT or IPT using a randomization generator sequence created by a statistician. Randomization was stratified by location (e.g., Maryland or Colorado) in blocks of 2–7 participants. Participants were continuously randomized until there was a complete cohort (approximately 10 adolescents, with 5 per group program) to run CBT and IPT groups in parallel. All of the group programs were facilitated by a psychologist, with support from a graduate student co-facilitator.

Before each weekly group session, participants completed a Treatment Process Questionnaire and the Mood Rating Checklist. At the end of Sessions three, six, and nine, the participants completed questionnaires to assess their negative and positive thoughts, anxiety-related behavior, relationship adjustment, and mood.

#### 2.3.5. Post-Treatment Assessment

Within approximately 2 weeks of the end of the 12-week group intervention, participants returned to the laboratory to repeat body measurements, blood draw, interviews, and questionnaires. They also wore the three ambulatory monitoring devices and completed the ecological momentary assessments for 7 days.

#### 2.3.6. The 1-Year Follow-Up

Approximately 1 year from the initiation of the group programs, participants repeated body measurements, blood draw, interviews, and questionnaires. They also wore the three ambulatory monitoring devices and completed the ecological momentary assessments for 7 days.

#### 2.3.7. The 2- and 3-Year Follow-Ups

Approximately 2- and 3 years, respectively, following the initiation of the group programs, participants repeat body measurements, interviews, and questionnaires. They do not re-wear monitors or complete ecological momentary assessments at these follow-ups.

### 2.4. Description of the Interventions

#### 2.4.1. General Intervention Structure and Format

Both the CBT and IPT programs met virtually for 12 weekly 90-min group sessions that occurred during after-school hours. Both of the programs also included one 90-min and two 30-min individual sessions that took place at the beginning, middle, and end of the 12-week group program, respectively. Group sessions were led by both facilitators, but the individual session could be led by one or both facilitators. The intervention was delivered only to the adolescents, except for a brief (i.e., 10–15 min) parent/guardian component in the 90-min individual session before the group program begins. During this brief joint segment, the facilitator met with the adolescent and parent/guardian together to review the guidelines for participation in the virtual format of group and to problem-solve with both the adolescent and parent/guardian potential barriers to engagement (e.g., access to a device with a working camera and microphone, having a private space).

In both of the programs, the adolescents were encouraged to apply the skills learned in the group sessions to address current anxiety-provoking situations that occurred in between sessions through home practice exercises, which were facilitated by a binder with handouts. At the beginning of each group session, the adolescents were invited to review the material learned in the previous group session and reflect on their experience doing the at-home practice exercises. Adolescents were reinforced for participation in-session and use of skills outside of session with rewards that were mailed at the mid- and endpoint of the 12-week group program. These rewards consisted of small items such as stickers, pens, hand sanitizer, and stress relief toys with a total value between $5 and $10.

All of the group facilitators met virtually for weekly supervision to discuss feedback on audio-recorded sessions. The group leaders were trained and supervised by clinical psychologists with expertise in CBT and IPT.

#### 2.4.2. Cognitive Behavioral Therapy (CBT)

The CBT program was adapted from the C.A.T. Project, a 16-session individual CBT program for anxiety in adolescents [107]. This program is based on the cognitive behavioral model, which posits that negative thoughts and avoidance behaviors can worsen or maintain negative affect, including anxiety. Anxiety symptoms in turn are known to be related to disinhibited eating. The program was adapted by the co-investigators/clinical psychologists (LDG, MHN) to match the general group format and structure of IPT. The main adaptations of the CBT program included adjusting language to address a group instead of individuals, decreasing group sessions to cover material in 12 versus 16 group sessions, adding adolescent individual sessions to the beginning, middle, and end of the program, and removing the parent individual sessions. The adapted CBT program also included psychoeducation about the connection between thoughts, behaviors, emotions, and disinhibited eating, and incorporated these connections in the application of cognitive and behavioral skills to cope with anxiety-provoking situations that were hypothesized to also impact on eating behavior.

Following the brief segment with the parent/guardian, the 90-min individual session continued with psychoeducation on the cognitive behavioral model and rationale and nature of CBT. The facilitator conducted a semi-structured clinical interview (i.e., a functional assessment of anxiety) to assess the adolescent’s feelings, thoughts, and behaviors in anxiety-provoking situations. The facilitator and adolescent collaboratively set one or two program goals to improve coping with select current anxiety-provoking situations throughout the 12-week group program.

As outlined in Table 2, Sessions 1–6 of the 12-week group program focused on recognizing signs of anxiety and learning cognitive-behavioral coping strategies to manage anxiety-provoking situations. For example, participants learned to detect physiological reactions and emotions and to manage these feelings with relaxation exercises, such as diaphragmatic breathing and progressive muscle relaxation. The participants also practiced identifying negative thoughts patterns and restructuring these thoughts to generate positive coping thoughts. Participants were encouraged to reward themselves for trying to apply these new coping skills in anxiety-provoking situations. Sessions 7–11 focused on in-session application of the cognitive-behavioral coping strategies during brief exposures that were meant to prepare for the application of these skills in current anxiety-provoking situations in between sessions. Finally, Session 12 was a termination session during which the group reviewed the impact of changes in cognitive and behavioral coping strategies, anxiety, and eating behaviors. Group members were encouraged to continue cognitive behavioral work on their own after the program concluded.

#### 2.4.3. Interpersonal Psychotherapy (IPT)

The IPT program [62] is adapted from group-based IPT-Adolescent Skills Training for the prevention of adolescent depression [108] and group-based IPT for the treatment of binge-eating disorder [109]. This IPT program is adapted specifically for adolescent girls at risk for excess weight gain due to BMI and disinhibited eating behaviors. IPT is based on the interpersonal theoretical model, which posits that low social support and high conflict can worsen or maintain negative affect, including anxiety. Negative affect, in turn, is believed to promote disinhibited eating [72]. IPT therefore includes psychoeducation about the connection between social relationships, mood, and eating behavior, as well as teaching communication and interpersonal problem-solving skills to manage conflict and increase social support.

Following the brief segment with the parent/guardian, the 90-min individual session continues with psychoeducation on the interpersonal theoretical model and rationale and nature of IPT. The facilitator conducts a semi-structured clinical interview called an “interpersonal inventory” to assess the adolescent’s current relationships, with an explicit focus on linking the adolescent’s relationships to anxiety and current disinhibited eating [110]. Based on IPT for a binge-eating disorder, the facilitator creates a timeline linking major life events to episodes of disinhibited eating [109]. Finally, the facilitator and adolescent collaboratively set one or two program goals to address support and conflict in current relationships throughout the 12-week group program.

As described in Table 3, Sessions 1–3 of the 12-week group program comprised the initial phase of IPT. Facilitators focused on building rapport and commonalities among adolescents. In addition to reviewing psychoeducation on the interpersonal theoretical model and rationale and nature of IPT, the adolescents learned communication analysis to identify the impact of verbal and nonverbal communication on emotions and on the “back-and-forth” of a conversation. Adolescents also learned seven communication skills (e.g., finding the right time to have a conversation, labeling emotions, acknowledging another’s point of view, etc.). Sessions 4–9 comprise the middle phases of IPT, in which the facilitators helped the adolescents to role-play communication skills in-session to prepare for the application of these skills in current relationships in between sessions. Sessions 10–12 comprised the termination phase, during which the group reviewed the impact of changes in communication on support/conflict in current relationships, anxiety, and eating behaviors. Session 12 also included a review of warning signs of worsening anxiety or disinhibited eating behaviors, as well as identifying when and how to seek help. Group members were encouraged to continue the interpersonal work on their own after the group program concluded.

## 3. Results

### 3.1. Feasibility Indicators

Feasibility of recruitment was operationalized as enrollment of the target sample size (*N* = 40; *n* = 20 per site) within a 12-month period. We were also calculating the percentage of youth, estimated to be eligible, who decide to enroll. All of the recruitment methods and responses were tracked by site according to the CONSORT guidelines for pilot studies [111].

Feasibility of the study protocol was determined by retention at each follow-up interval, with benchmarks of 80% or more retention at post-treatment and 70% or more retention at 1-year, 2-year, and 3-year follow-ups. Additionally, we expected <5% data missingness for survey, interview, laboratory, and blood measures that were administered in the lab, 80% or more adherence to ambulatory device wear (see below) to assess daily glucose patterns, heart rate variability, and sedentary time, and 80% adherence to ecological momentary assessments (see below). Research staffs’ compliance with standardized operating procedures was determined through checklists for all assessments, and the benchmark was at least 95% quality assurance. Percentages and reasons for missing data were reported.

Multisite intervention fidelity, an indicator of the feasibility of intervention training and delivery, was assessed by review of 20% of the audio-recorded CBT and IPT group sessions. Ratings were completed by blinded and independent, trained raters using structured checklists to assess adherence to the respective manuals.

### 3.2. Acceptability Indicators

The acceptability of CBT and IPT group interventions delivered via telehealth was evaluated using several metrics. We expected 80% or more of the participants to receive at least 80% of the dose (i.e., attend at least 10 of 12 sessions). Participants completed an adapted post-treatment acceptability questionnaire [112] to rate likability and credibility of the programs on a scale of “1” (not at all or much worse) to “5” (extremely, definitely, or much better), with “5” being the most positive valence. Examples of questions measuring likeability include “If you could do it over, how likely would you be to take part in the group program?” and “How much did you enjoy coming to the group program?” Questions measuring credibility include “How helpful do you think the group program would be to other people your age?” and “Were the group program leaders supportive?” Acceptability was determined as an average rating of “4” or higher. Before the intervention, we administered the Treatment Expectancy Questionnaire which consists of 10 questions that measure which group condition participants would prefer if they had the choice and to what extent they anticipate the group will improve their mood, stress, and health. Participant preference and expectation are both components of intervention acceptability and have been shown to relate to intervention outcomes [113].

The acceptability of ambulatory device wear and ecological momentary assessments were assessed to determine any necessary adjustments for a larger future study. Acceptability of ambulatory device wear and ecological momentary assessments were assessed with a survey asking adolescents to rate on a 5-point scale from “1” (not at all) to “5” (extremely) the comfort, ease, and convenience of these real-time, real-world measurement approaches.

## 4. Discussion

The current study is a multisite pilot and feasibility randomized controlled trial of group CBT and IPT, delivered via telehealth, for adolescent girls with elevated anxiety symptoms who are at risk for excess weight gain. The primary aims of the multisite study are to evaluate feasibility of recruitment, feasibility of the study protocol and data collection, intervention fidelity, retention to follow-up, and acceptability of interventions and study participation. Results will directly inform the design of a future randomized controlled trial to test the efficacy of group telehealth CBT and IPT for preventing excess weight gain and the worsening of cardiometabolic health, as well as theoretically informed treatment moderators and mediators.

Preventative interventions for excess weight gain among adolescents that focus directly on healthy eating and physical activity have not been shown to be efficacious [15,16]. Alternatively, interventions targeting specific, theory-informed vulnerabilities underlying disinhibited eating may offer a more effective and enduring approach to prevent excess weight gain and cardiometabolic health problems. Anxiety in particular appears to be a promising treatment target among adolescent girls [37,114] and has been implicated in the onset and maintenance of disinhibited eating in youth [24,35]. Both CBT and IPT show promise for reducing anxiety symptoms and disinhibited eating, as well as some initial positive signals for ameliorating weight-related and cardiometabolic health trajectories among adolescent girls with symptoms of anxiety or depression and at risk for excess weight gain [56]. However, no study to date has compared group CBT and IPT in ameliorating excess weight gain trajectories among this population of adolescent girls, nor simultaneously investigated the mechanisms through which the interventions uniquely target anxiety and disinhibited eating.

This multisite pilot and feasibility randomized controlled trial is a necessary step in preparing for a larger efficacy trial. Information about recruitment rate and yields from various methods across sites will directly inform the recruitment plan and timeline for the efficacy trial. The assessments are relatively high in volume, involving body measurements, questionnaires, interviews, blood draws, ambulatory device wear, and ecological momentary assessments. The assessment plan was designed to determine the multisite feasibility of the study protocol and data collection, as a comprehensive battery of this kind will be needed to fully characterize the potential mechanistic factors underlying anxiety and health outcomes in the future efficacy trial. There appears to be considerable added value to including real-time, real-world measures of heart rate, glucose, physical activity, affect, eating behaviors, cognitions, behaviors, and social interactions, particularly with regard to ecological validity and the potential to characterize intraindividual changes in and interactions between these variables [115]. Participant acceptability ratings for the real-time, real-world monitoring devices and ecological momentary assessments will be especially useful for informing whether they should be included in a future efficacy trial.

The characterization of the intervention fidelity and acceptability of CBT and IPT interventions delivered in a group format via telehealth are very important to this pilot and feasibility study. Although telehealth delivery offers many advantages, such as widespread accessibility, increased attendance, and reduced cost of care [116,117], it is important to establish its acceptability to adolescent girls with elevated anxiety symptoms who are at risk for excess weight gain. It is possible that girls will feel more, or perhaps less, comfortable compared to in-person group therapy. Although this pilot study was not designed to compare telehealth versus in-person delivery, average ratings of group telehealth CBT and IPT can be compared to ratings from previous, similar intervention studies to gauge relative acceptability. Likewise, the establishment of strong fidelity to the CBT and IPT manuals is particularly important for honing training plans and/or intervention delivery processes for a future trial comparing two active interventions to ensure robust fidelity and minimal overlap.

We will not statistically compare the efficacy of CBT and IPT for clinical and health outcomes in this pilot study. However, describing change by condition and variability in changes in outcomes that would be tested in a future study will be informative and offer guidance in finalizing the selection of primary and secondary outcomes for the larger trial. Within the CBT group, we may observe a signal for improvements in the thoughts and behaviors that are targeted to reduce anxiety. Within the IPT group, we may observe a signal for improved social functioning, which is the primary target of IPT. In a future efficacy trial, we do not necessarily anticipate significant differences in efficacy for distal outcomes, such as BMI, adiposity, and cardiometabolic health outcomes. However, investigating the differential action mechanisms through which CBT and IPT work, as well as factors that may moderate treatment outcomes, is critical to facilitate precise prevention approaches to obesity and cardiometabolic disease [16]. Given the dearth of effective prevention and treatment options among adolescents, it is imperative to develop targeted preventative approaches to prevent serious long-term health consequences.

## Figures and Tables

**Table 1:** Overview of a participant timeline, assessment intervals, and core measures.

Construct: Assessment	Baseline		Post-Treatment	1-Year	2- and 3-Year
Informed consent/assent	X	**Randomization: CBT or IPT** **12-week Intervention Group**			
Demographics questionnaire	X				
At-home device acceptability questionnaire	X		X	X	
Treatment expectation questionnaire	X				
Intervention acceptability questionnaire			X		
**Weight and body composition**					
BMI indices: Height and weight	X		X	X	X
Adiposity: Bod Pod measurement, waist circumference	X		X	X	X
**Cardiometabolic health**					
Insulin resistance, glucose, lipids, and A1c: Fasting blood draw	X		X	X	X
Glycemic variability: CGM *	X		X	X	
Resting blood pressure: Automatic blood pressure monitor	X		X	X	X
**Stress-related physiology**					
Heart rate and heart rate variability: CAM *	X		X	X	
**Disinhibited eating**					
Eating in absence of hunger: EES-C, EAH-C-PR	X		X	X	X
Loss-of-control eating: EDE-O, EDA-5, EMA *	X		X	X	X
**Internalizing symptoms**					
Anxiety symptoms: SPAI-C, EMA *	X		X	X	X
Depression symptoms: CES-D, EMA *	X		X	X	X
**Cognitive and behavioral functioning**					
Negative and positive thoughts: CATS-N/P, EMA *	X		X	X	X
Healthy coping and anxiety control: CCSC-R1, ACQ-C, SEQ-C	X		X	X	X
**Social functioning**					
Social support and adjustment: NRI-Short Form, SAS	X		X	X	X
Conflict resolution: CBQ-20	X		X	X	X
**Other Outcomes**					
Daily physical activity: activPAL *	X		X	X	
Affect and emotion regulation: PANAS-C, DERS-SF, EMA *	X		X	X	X

**ACQ-C** survey: Anxiety Control Questionnaire for Children; **BMI**: body mass index indices derived from height and weight; **CAM:** Carnation ambulatory monitor; **CATS-N/P** survey: Children’s Automatic Thoughts Scale—Negative/Positive (CATS-N/P); **CBT**: cognitive behavioral therapy; **CBQ-20** survey: Conflict Behavior Questionnaire; **CCSC-R1** survey: Children’s Coping Strategies Checklist-Revised; **CES-D** survey: Center for Epidemiologic Studies-Depression Scale **CGM**: continuous glucose monitor; **DERS-SF** survey: Difficulties in Emotion Regulation Scale-Short Form **EAH-C-PR** survey: Eating in the Absence of Hunger Questionnaire for Children-Parent Report; **EDA-5** interview: Eating Disorder Assessment for DSM-5; **EDE-O** interview: The Eating Disorder Examination-Overeating Section; **EES-C** survey: Emotional Eating Scale-Children; **EMA**: ecological momentary assessment; **IPT**: interpersonal therapy; **NRI** survey: Network of Relationships Inventory-Short Form; **PANAS-C** survey: Positive and Negative Affect Schedule-Child Form; **SAS** survey: Social Adjustment Scale-Self Report; **SEC-Q** survey: Self-Efficacy Questionnaire for Children; **SPAI-C** survey: Social Phobia and Anxiety Inventory for Children. * EMA and devices (CGM, CAM, activPAL) not collected at 2- and 3-year follow-ups.

**Table 2:** Summary of CBT session content.

Session	Content
Pre-	Review feelings/thoughts/behaviors associated with participant’s own anxiety; set individual goals
1	Rules and purpose of group; psychoeducation about thoughts/behaviors, anxiety, disinhibited eating
2	Recognize emotions in self and others; learn somatic responses to anxiety in general
3	Identify individual’s own somatic responses to anxiety; introduce relaxation exercises
4	Recognize negative thoughts, challenge their utility and accuracy, and replace with coping thoughts
5	Learn problem-solving to actively cope with anxiety-related feelings, thoughts, and avoidance
6	Conduct self-evaluation and self-reinforcement for active coping with rewards
Mid-	Review progress towards individual goals and make a plan for individual work for rest of group
7	Discuss coping with hypothetical anxiety-provoking scenarios; introduce in-session exposures
8–10	Participants each plan and complete 10-min in-session exposure with facilitator/group support
11	Continue exposures; introduce participant presentations at Session 12 to review what they learned
12	Complete exposures; presentations; review, practice, maintain, and generalize skills; graduation
Post-	Review progress towards goals and make a plan for individual work now that group has ended

**Table 3:** Summary of IPT session content.

Session	Content
Pre-	Review relationship issues associated with participant’s own anxiety; set individual goals
1	Rules and purpose of group; psychoeducation about conflict/support, anxiety, disinhibited eating
2	Practice communication analysis to understand impact of verbal/non-verbal communication
3	Learn communication skills to decrease conflict and increase support
4	Introduce in-session role-play to practice using communication skills
5–6	Continue discussion, role play, and problem solving from participants’ current relationships
Mid-	Review progress towards individual goals and make a plan for individual work for rest of group
7–9	Continue discussion, role play, and problem solving from participants’ current relationships
10, 11	Identify useful communication strategies and discuss barriers and solutions to generalize skills
12	Recognize characteristics of positive/healthy relationships; maintaining skills; graduation
Post-	Review progress towards goals and make a plan for individual work now that group has ended

## Data Availability

Not applicable.
